# A systematic review and meta-analysis of cemented and uncemented bipolar hemiarthroplasty for the treatment of femoral neck fractures in elderly patients over 60 years old

**DOI:** 10.3389/fmed.2023.1085485

**Published:** 2023-02-02

**Authors:** Mengyu Fu, Jieliang Shen, Zhoukui Ren, Yingwen Lv, Jiangang Wang, Wei Jiang

**Affiliations:** ^1^Department of Orthopaedics, The Thirteenth People’s Hospital of Chongqing (The Geriatric Hospital of Chongqing), Chongqing, China; ^2^Department of Orthopaedics, The First Affiliated Hospital of Chongqing Medical University, Chongqing, China

**Keywords:** femoral neck fractures, elderly patients, cemented, uncemented, bipolar hemiarthroplasty, meta-analysis

## Abstract

**Background:**

Currently, whether bone cement can be applied in bipolar hemiarthroplasty to treat femoral neck fractures (FNFs) in elderly patients is controversial. The aim of this systematic review and meta-analysis was to compare the effectiveness and safety of cemented bipolar hemiarthroplasty (CBH) versus uncemented bipolar hemiarthroplasty (UCBH) in the treatment of FNFs among elderly patients over 60 years old.

**Materials and methods:**

The Pubmed, Web of science, Cochrane Library and EMBASE databases were searched comprehensively for relevant articles from their inception to May 2022. Studies about comparing outcomes between CBH and UCBH for FNFs in elderly patients aged more than 60 years were included. Outcomes including operation time, intra-operative blood loss, length of hospital stay, wound infections, residual pain, revisions, re-operations, complications related to prosthesis, general complications, and mortality. The Review Manager 5.3 software provided by the Cochrane Collaboration Network was used to perform the meta-analysis of comparable data.

**Results:**

A total of 6 randomized controlled trials (RCTs) and 9 observational studies were included in this analysis, with 33,118 patients (33,127 hips). Results of the meta-analysis indicated that the operation time [WMD = 13.01 min, 95% CI (10.79, 15.23)], intra-operative blood loss [WMD = 80.57 ml, 95% CI (61.14, 99.99)], incidence of heterotrophic ossification [OR = 2.07, 95% CI (1,14, 3.78)], were increased in the CBH group but the incidence of intra-operative fractures [OR = 0.24, 95% CI (0.07, 0.86)], periprosthetic fractures [OR = 0.24, 95% CI (0.18, 0.31)], aseptic loosening of prosthesis [OR = 0.20, 95% CI (0.09, 0.44)], wound infections [OR = 0.80, 95% CI (0.68, 0.95)] and re-operation rates [OR = 0.61, 95% CI (0.54, 0.68)] were lower in the CBH group by comparison with the UCHB group. However, there were no significant differences in residual pain, length of hospital stay, prosthetic dislocation, prosthetic subsidence (> 5 mm), acetabulum erosion, revisions, pulmonary infections, pulmonary embolisms, urinary tract infections, deep venous thromboses, decubitus, cardiovascular accidents (arrhythmia/myocardial infarction), and respiratory failure between the two groups. In terms of mortality, perioperative mortality (within 72 h) [OR = 2.39, 95% CI (1.71, 3.32)] and 1-week mortality postoperatively [OR = 1.22, 95% CI (1.05, 1.41)] in CBH group were higher than those in UCBH group, but there were no significant differences in mortality at 1 month, 3 months, 1 year, and 2 years postoperatively between CBH group and UCBH group.

**Conclusion:**

This meta-analysis found that elderly patients over 60 years old with FNFs who underwent CBH had longer operation time, higher incidence of heterotrophic ossification, intra-operative blood loss, and mortality within 72 h of operation and at 1-week postoperatively, but lower incidence of periprosthetic fractures, aseptic loosening of prosthesis, intra-operative fractures, wound infections and re-operations. Other outcomes were not significantly different between the two groups.

**Systematic review registration:**

https://www.crd.york.ac.uk/PROSPERO/, identifier CRD42021274253

## 1. Introduction

Due to an aging population, the annual incidence of osteoporosis is on the rise and these are often complicated by concomitant hip fractures (1). Hip fractures—whose incidence increases with age—are not only common in the elderly (2) but are also predominated—approximately 50%—by femoral neck fractures (FNFs) (3). Due to anatomical reasons, the femoral head becomes insufficiently supplied with blood after an FNF, which then leads to femoral head necrosis and bone non-union (4). Moreover, non-operative treatment requires long-term bed rest that often leads to various complications, and the 30-day mortality is between 5 and 10% (3). The standard treatment of elderly patients with FNFs is arthroplasty, which can achieve good results such as improved hip function, relief of hip pain, early ambulation, and return to independent living (5). Compared to total hip arthroplasty, hemiarthroplasty has the advantage of less trauma, shorter operation durations, less bleeding, and lower cost. This is suitable for elderly patients who generally are in a poor condition and unable to tolerate major surgery (6). Bipolar hemiarthroplasty is more commonly used in clinics than unipolar hemiarthroplasty, as the bipolar head increases the intra-articular mobility and reduces the relative movement between the prosthesis and acetabular cartilage and subchondral bone, which is expected to reduce the wear of acetabulum and prolong the life of the prosthesis (6, 7). Relatedly, elderly patients with FNFs who underwent bipolar hemiarthroplasty achieved a good clinical outcome, with a high rate of return to the pre-injury state, a great range of hip motion, and fast walking speed (8).

Today, the use of bone cement in bipolar hemiarthroplasty for the treatment of FNFs in elderly patients remains debatable (9, 10). Traditionally, cemented prostheses were mainly for elderly patients with poor bone quality (11). Indeed, as per the 2016 Dutch Orthopedic Association (DOV) guidelines, the preferred way to treat elderly patients with displaced FNFs is cemented hemiarthroplasty (12). Although cemented hemiarthroplasty results in better implant fixation, less implant-related complications, and better functional outcomes (13, 14), it is associated with a high risk of cardiovascular and respiratory complications such as myocardial infarction/arrhythmias and cardio-respiratory collapse due to cement-related toxicity and embolization of bone cement (15–17). On the other hand, uncemented hemiarthroplasty has short operation durations and low blood loss (18). Moreover, its use avoids bone cement implantation syndrome (BCIS) which increased perioperative mortalities (3)—the incidence of BCIS in cemented hemiarthroplasty ranged from 28 to 72% (16, 19). Due to variations in countries and regions, patient ethnic groups, and surgical techniques of operators, there is no consensus on these issues.

Most previous systematic reviews and meta-analyses on the comparison of cemented and uncemented hemiarthroplasty in the treatment of hip fractures included both unipolar and bipolar implants. Hence, this study aimed to comprehensively compare the effectiveness and safety of cemented bipolar hemiarthroplasty (CBH) and uncemented bipolar hemiarthroplasty (UCBH) for the treatment of FNFs in patients older than 60 years of age, *via* assessments of operation time, intra-operative blood loss, length of hospital stay, residual pain, re-operations, revisions, wound infections, prosthesis-related complications, general complications, and mortality.

## 2. Materials and methods

### 2.1. Search strategy

This study was designed and conducted according to the Preferred Reporting Items for Systematic Reviews and Meta-Analyses (PRISMA) guidelines (20). Two reviewers independently performed electronic searches of the PubMed, Web of Science, EMBASE, and Cochrane Library database up to May 2022. The search consisted of terms relating to the condition including “hemiarthroplasty,” “hemiprosthesis,” “hemiarthroplasties,” “replacement,” “arthroplasty,” “artificial femoral head replacement,” “artificial femoral head arthroplasty,” “bipolar,” “cement,” “cemented,” “uncement,” “uncemented,” “cementless,” “without bone cement,” “non-cemented” (Supplementary Table 1). Moreover, we assessed the reference lists of relevant reviews to identify additional relevant studies. Any disagreements were resolved by a third author. The search was not restricted by language nor publication date. Finally, the systematic review and meta-analysis was registered on the PROSPERO website under the registration number CRD42021274253.

### 2.2. Inclusion and exclusion criteria

We enrolled randomized clinical trials (RCTs) and observational studies that met the following criteria: (1) the patients with FNFs were older than 60 years of age; (2) the intervention measures were either CBH or UCBH; (3) at least an outcome was reported and used for meta-analysis; (4) the presence of a comparison of outcomes between CBH and UCBH; (5) the full text was available. We excluded studies based on the following criteria: (1) the patients were younger than 60 years of age; (2) they were reviews, case reports, duplicates, conference abstracts; (3) they were basic research on animals or cadavers; (4) non-controlled studies; (5) we were unable to obtain the original text or extract accurate data; (6) they studied patients with a previous fracture in the same hip; (7) the patients with other pathological FNFs, such as tuberculosis, tumor, infection, and metabolic osteopathy.

### 2.3. Literature review

Two researchers independently searched for literature using the keywords and used the Endnote Document Management software to not only eliminate duplicated studies but also extract valid information *via* the literature titles and abstracts. After excluding articles as per the criteria, full texts were read to determine whether they met the inclusion criteria. Resultant studies were cross-checked and disagreements would be solved through discussion or decided by a third but senior researcher.

### 2.4. Data extraction

We extracted information using a pre-designed data extraction table. The following information was extracted by two independent researchers from the included studies: first author, publication date, country, study design, sample size, mean age, gender, surgical approach, type of intervention, type of prosthesis, American Society of Anesthesiologists (ASA) grade, follow-up period, and relevant clinical outcomes.

### 2.5. Outcome measures

We included studies reporting at least one of the following outcomes: operation time, intra-operative blood loss, length of hospital stay, residual pain, re-operation, revision, wound infection, mortality (perioperative period, postoperative a week, a month, 3 months, a year, and 2 years), complications related to prostheses such as dislocation, heterotrophic ossification, periprosthetic fracture, aseptic loosening of the prosthesis, subsidence of prosthesis (> 5 mm), intra-operative fracture and acetabulum erosion, general complications such as postoperative pulmonary infection, pulmonary embolism, urinary tract infection, deep venous thrombosis, decubitus, cardiovascular accidents (arrhythmia/myocardial infarction), and respiratory failure.

### 2.6. Quality assessment

Two researchers independently evaluated the quality of the RCTs and observational studies—disagreements were solved either through discussion or decided by the third senior researcher. The Cochrane Risk of Bias (ROB) assessment tool (21) was used to assess the quality of each RCT. The risk of bias was assessed from random sequence generation, concealment of the allocation sequence, blinding of participants and personnel, blinding of outcome assessment, incomplete outcome, and selective reporting. The assessment for each entry involved answering a question, with either “yes”—indicating low risk of bias—or “No”—indicating high risk of bias—or “unclear”—indicating lack of information/uncertainty over the potential for bias (21). If all items were assessed with “Yes” then the quality grade was “A” which meant high quality. If one or more items were evaluated as “unclear” then the quality grade was “B” which meant moderate quality. If one or more items were evaluated as “No” then the quality grade was “C” which meant low quality. The Newcastle Ottawa scale (NOS) (22) was used to assess the quality of each observational study. Here, the risk of bias was assessed based on three essential domains which contained eight items (22): selection of the study subjects (4 points), comparability of groups (2 points), and ascertainment of the exposure or outcome (3 points). The highest score was 9, with higher scores indicating higher quality of included studies. Studies were classed as of high, moderate, and low quality when total scores were ≥ 7, 4–6 and ≤ 3 points, respectively.

### 2.7. Data synthesis and analysis

The Review Manager 5.3 software provided by the Cochrane Collaboration Network was used to perform a meta-analysis of comparable data. We calculated odds ratios (ORs) with a 95% confidence interval (CI) for dichotomous outcomes and weighted mean difference (WMD) with a 95% CI for continuous data. The heterogeneity among included studies was tested using the chi-square (χ^2^) test and I-square (*I*^2^) tests (23), and *P*-value > 0.10 and an *I*^2^ ≤ 50% was considered insignificant heterogeneity. The fixed-effect model was used when there was insignificant heterogeneity between studies (*P* > 0.10, *I*^2^ ≤ 50%). Conversely, the random-effect model was used when significant heterogeneity existed between studies (*P* < 0.10, *I*^2^ > 50%). Moreover, further sensitivity analyses were performed to investigate the potential origin of heterogeneity. Sensitivity analysis was conducted by omitting a study at a time and pooling the data from the remaining studies to explore possible sources of the high heterogeneity and determine the stability of the outcomes (9). Egger’s test or funnel plots were used to estimate publication bias when 10 or more studies were presented (24). The funnel plots were drawn in Review Manager 5.3 software and the Egger’s test was performed in STATA 12 software at an alpha level of *P* < 0.05.

## 3. Results

### 3.1. Demographics and characteristics

**FIGURE 1:**
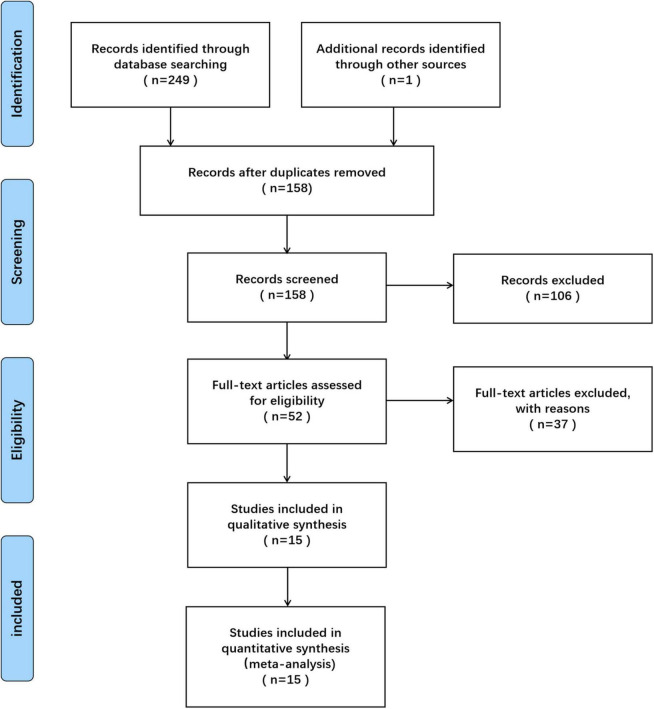
Preferred reporting items for systematic reviews and meta-analyses (PRISMA) flow diagram of study selection.

249 articles were obtained in the preliminary election, 157 repeatedly published articles were eliminated by Endnote Document Management software, and 106 articles that obviously did not meet the inclusion criteria were eliminated after reading the title and abstract. After reading the full text of included studies and carrying out quality assessment, 37 unqualified articles were further removed, and 1 qualified literature was obtained by manual retrieval *via* assessing the reference lists of relevant reviews. Finally, 15 articles were found to be eligible and included in the meta-analysis (25–39). Among them, 6 were RCTs (25–30) and 9 were observational studies (31–39). The PRISMA flow diagram showing the study selection process is shown in Figure 1.

Notably, there were 33,118 patients (33,127 hips) over 60 years old in the 15 included studies, which comprised 24,074 cases who underwent CBH and 9,053 cases who underwent UCBH. The characteristics of the included studies are summarized in Tables 1, 2. Although the study conducted by Langslet E et al. (26) was the same to that conducted by Figved W et al. (28), both studies were included because the follow-up time in the two studies was different, suggesting that there were some outcomes were different to be included. The follow-up time of the study conducted by Figved W et al. (28) was 12 months, however the follow-up time of the study conducted by Langslet E et al. (26) was prolonged to 60 months, therefore there was no duplicated data included in this meta-analysis. In addition, the number of patients was inconsistent with the number of operated hips in three studies (26, 28, 39) because some patients underwent bipolar hemiarthroplasty in both left and right hips at different time-points. Therefore, the mortality was calculated according to the number of patients whereas other outcomes were calculated according to the number of operated hips.

**TABLE 1:** Baseline characteristics of RCTs included in review.

References	Country	Study type	Study period	Garden grade	Approach	Number of patients		Number of hips		Mean age (year)		Gender (numbers)	
						CHA	UCHA	CHA	UCHA	CHA	UCHA	CHA	
												Male	Female
Movrin I (25)	Slovenia	RCT	2013.01–2015.12	III, IV	Standard anterolateral approach	79	79	79	79	86 ± 5 (≥ 76)	84 ± 4 (≥ 76)	33	46
Langslet E et al. (26)	Norway	RCT	2004.09–2006.08	-	Posterior approach	108	105	112	108	83.4 ± 5.68 (≥ 70)	83.0 ± 6.29 (≥ 70)	25	87
Talsnes O et al. (27)	Norway	RCT	2005–2010	III, IV	-	162	172	162	172	84.3 ± 5.0 (≥ 75)	84.0 ± 5.1 (≥ 75)	45	117
Figved W et al. (28)	Norway	RCT	2004.09–2006.08	-	Posterior approach	108	105	112	108	83.4 ± 5.68 (≥ 70)	83.0 ± 6.29 (≥ 70)	25	87
Santini S et al. (29)	Italy	RCT	2000.09–2001.12	-	Lateral approach	53	53	53	53	82.09 ± 7.60 (≥ 65)	79.68 ± 8.62 (≥ 65)	13	40
Emery RJ et al. (30)	UK	RCT	-	-	-	27	26	27	26	78 ± 7.2 (63–90)	79.6 ± 8 (61–96)	3	24

References	Country	Study type	Study period	Garden grade	Approach	Gender (numbers)		ASA (numbers)				Implant type		Follow-up	
						UCHA		CHA		UCHA		CHA	UCHA	CHA	UCHA
						Male	Female	I, II	III, IV	I, II	III, IV				
Movrin I (25)	Slovenia	RCT	2013.01–2015.12	III, IV	Standard anterolateral approach	31	48	40	39	46	33	Ecofit*™*	Ecofit*™*	3, 6, 12, and 24 months	3, 6, 12, and 24 months
Langslet E et al. (26)	Norway	RCT	2004.09–2006.08	-	Posterior approach	28	80	77	35	76	32	Spectron*™*; Smith and Nephew, Memphis, TN, USA	Corail*™*; DePuy/ Johnson and Johnson, Leeds, UK	60 months	60 months
Talsnes O et al. (27)	Norway	RCT	2005–2010	III, IV	-	37	135	68	94	68	104	Landos Titan, Depuy, Warshaw, IN, USA	Landos Corail, Depuy, Warshaw, IN, USA	12 months	12 months
Figved W et al. (28)	Norway	RCT	2004.09–2006.08	-	Posterior approach	28	80	77	35	76	32	Spectron*™*; Smith and Nephew, Memphis, TN, USA	Corail*™*; DePuy/ Johnson and Johnson, Leeds, UK	1 week; 1, 3, 12, and 24 months	1 week; 1, 3, 12, and 24 months
Santini S et al. (29)	Italy	RCT	2000.09–2001.12	-	Lateral approach	11	42	22	31	26	27	–	-	6 and 12 months	6 and 12 months
Emery RJ et al. (30)	UK	RCT	-	-	-	4	22	-	-	-	-	Thompson, Johnson and Johnson, England	Austin Moore, Johnson and Johnson, England	Mean: 17 months Range: 12 to 27 months	Mean: 18 months Range: 12 to 30 months

RCT, randomized controlled trial; CHA, cemented bipolar hemiarthroplasty; UCHA, uncemented bipolar hemiarthroplasty; ASA, American Society of Anesthesiologists.

**TABLE 2:** Baseline characteristics of observational studies included in review.

References	Country	Study type	Study period	Garden grade	Approach	Number of patients		Number of hips		Mean age (year)		Gender (numbers)	
						CHA	UCHA	CHA	UCHA	CHA	UCHA	CHA	
												Male	Female
Kristensen TB et al. (31)	Norway	Observational study	2005.01–2017.12	III, IV	Anterior, lateral, and posterior approach	22639	7539	22639	7539	84 ± 6 (≥ 70)	84 ± 6 (≥ 70)	6339	16300
Song JSA et al. (32)	Canada	Observational study	2010.01–2016.05	-	-	361	296	361	296	81.4 (≥ 65)	80.3 (≥ 65)	88	273
Rai SK et al. (33)	Indian	Observational study	2013.01–2015.07	-	posterior approach	42	42	42	42	79.5 ± 5.04 (≥ 65)	75.9 ± 4.04 (≥ 65)	16	26
Choi JY et al. (34)	Korea	Observational study	2009.03–2015.02	III, IV	Modified Hardinge approach	115	65	115	65	77 (≥ 65)	76 (≥ 65)	31	84
Khorami M et al. (35)	Iran	Observational study	2011.01–2015	-	-	22	29	22	29	79 (70–92)	71.7 (65–76)	2	20
Cicek H et al. (36)	Turkey	Observational study	2007–2012	-	Posterolateral approach	43	41	43	41	75. ± 17.78 (≥ 70)	77.5 ± 13.75 (≥ 70)	20	23
Ng and Krishna (37)	Singapore	Observational study	2005.01–2009.12	-	-	96	111	96	111	73 (60–91)	72 (60–87)	21	75
Viberg B et al. (38)	Denmark	Observational study	1991–1998	-	-	209	360	209	360	83 (79–88)	84 (80–89)	40	169
Lo WH et al. (39)	China	Observational study	1985.10–1990.07	-	Anterolateral or Moore’s approach	113	131	114	132	65–93	62–94	-	-

References	Country	Study type	Study period	Garden grade	Approach	Gender (numbers)		ASA (numbers)				Implant type		Follow-up	
						UCHA		CHA		UCHA		CHA	UCHA	CHA	UCHA
						Male	Female	I, II	III, IV	I, II	III, IV				
Kristensen TB et al. (31)	Norway	Observational study	2005.01–2017.12	III, IV	Anterior, lateral, and posterior approach	2262	5277	7754	14885	2731	4808	Various types	Various types	Median: 24 months Interquartile range: 6 to 50.4 months	Median: 24 months Interquartile range: 6 to 50.4 months
Song JSA et al. (32)	Canada	Observational study	2010.01–2016.05	-	-	88	208	107	221	81	179	-	-	12 months	12 months
Rai SK et al. (33)	Indian	Observational study	2013.01–2015.07	-	posterior approach	19	23	-	-	-	-	Modular^®^	Modular^®^	Mean: 18.07 months Range: 6 to 30 months	Mean: 18.07 months Range: 6 to 30 months
Choi JY et al. (34)	Korea	Observational study	2009.03–2015.02	III, IV	Modified Hardinge approach	19	46	-	-	-	-	Various types	Various types	Mean: 28 months Arange: 6 to 73 months	Mean: 26 months Arange: 6 to 71 months
Khorami M et al. (35)	Iran	Observational study	2011.01–2015	-	-	17	12	-	-	-	-	Zimmer	Zimmer	Mean: 18.9 months Arange: ≥ 6 months	Mean: 19.5 months Arange: ≥ 6 months
Cicek H et al. (36)	Turkey	Observational study	2007–2012	-	Posterolateral approach	18	23	0	43	0	41	Corin^®^ TaperFit	Corin^®^ MetaFix	Mean: 44.8 months Range: 29 to 59 months	Mean: 47.4 months Range: 31 to 58 months
Ng and Krishna (37)	Singapore	Observational study	2005.01–2009.12	-	-	25	86	-	-	-	-	-	-	Mean: 28.8 months Range: 24 to 50.4 months	Mean: 28.8 months Range: 24 to 50.4 months
Viberg B et al. (38)	Denmark	Observational study	1991–1998	-	-	70	290	-	-	-	-	Ultima	Charnley-Hastings, Furlong	Range: 144 to 228 months	Range: 144 to 228 months
Lo WH et al. (39)	China	Observational study	1985.10–1990.07	-	Anterolateral or Moore’s approach	-	-	-	-	-	-	Bateman	Bateman	Mean: 34 months Range: ≥ 24 months	Mean: 34 months Range: ≥ 24 months

CHA, cemented bipolar hemiarthroplasty; UCHA, uncemented bipolar hemiarthroplasty; ASA, American Society of Anesthesiologists.

**FIGURE 2:**
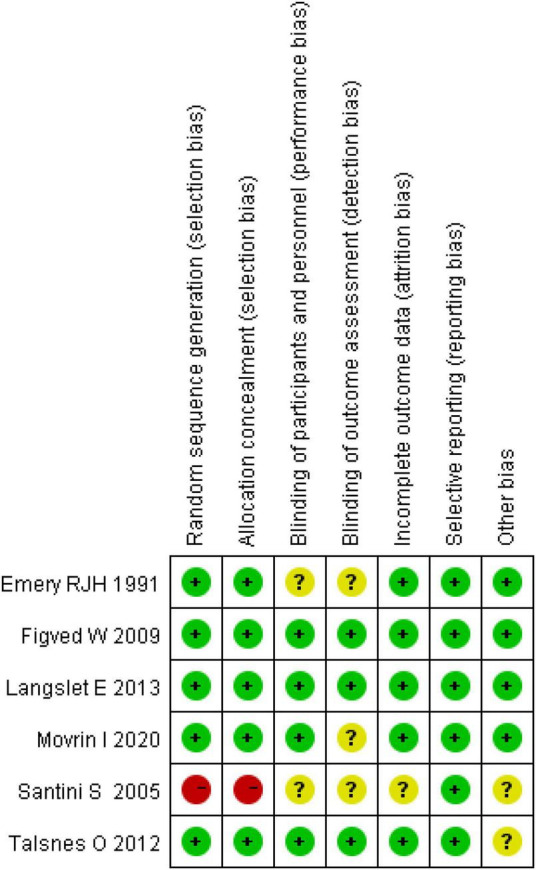
Risk of bias assessment of randomized controlled trials.

**FIGURE 3:**
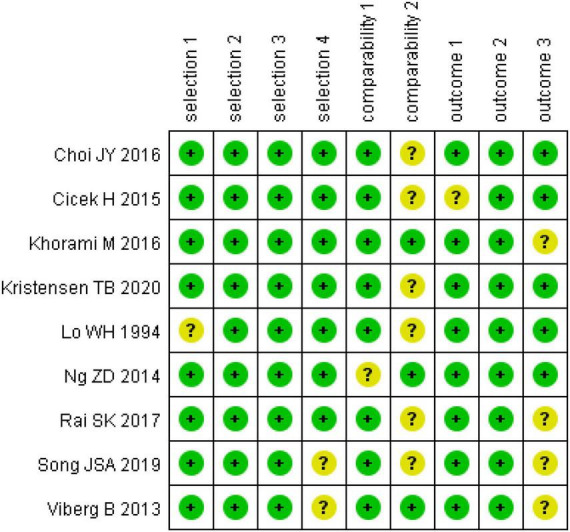
Risk of bias assessment of observational studies.

The Cochrane Collaboration’s tool was used to assess the risk of bias in RTCs (21). We found that two studies had a quality grade of “A” (26, 28), three were “B” (25, 27, 30) and one was “C” (29). According to the Newcastle-Ottawa scale (NOS) (22), eight studies were classed as high quality (total scores ≥ 7) (31, 33–39) and one study was classed as moderate quality (total scores between 4 and 6) (32). The summary of risk of bias scores in RCTs and observational studies are shown in Figures 2, 3 (Supplementary Tables 2, 3).

### 3.2. Meta-analysis results

#### 3.2.1. Operation time

**FIGURE 4:**
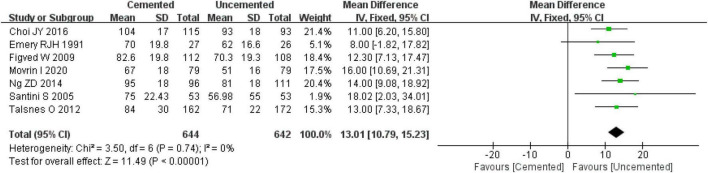
Forest plot for comparison of operation time between cemented bipolar hemiarthroplasty (CBH) group and uncemented bipolar hemiarthroplasty (UCBH) group.

A total of seven studies reported the operation time, among which five were RCTs (25, 27–30) and two were observational studies (34, 37). A total of 1,258 cases were included in the analysis, 644 underwent CBH and 642 underwent UCBH. There was no evidence of heterogeneity among the studies (*I*^2^ = 0%, *P* = 0.74). The fixed-effect model analysis revealed that the operation time was significantly shorter in the UCBH group relative to the CBH group [WMD = 13.01 min, 95% CI (10.79, 15.23), *P* < 0.00001; Figure 4].

#### 3.2.2. Intra-operative blood loss

**FIGURE 5:**
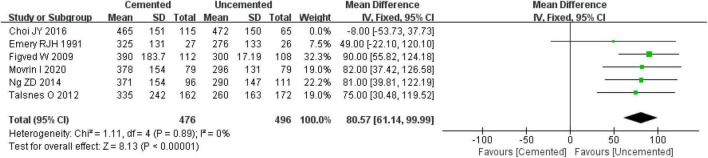
Forest plot for comparison of intra-operative blood loss between cemented bipolar hemiarthroplasty (CBH) group and uncemented bipolar hemiarthroplasty (UCBH) group.

Six studies reported intra-operative blood loss, including four RCTs (21, 27, 28, 30) and two observational studies (34, 37). Pooled effect size of the six studies revealed significantly high heterogeneity (*I*^2^ = 62%, *P* = 0.02), and a further sensitivity analysis indicated that the heterogeneity was caused by the study by Choi JY et al. (34). Thus, the study by Choi JY et al. (34) was excluded and then the fixed-effect model was employed to analyze the remaining studies. It was found that intra-operative blood loss was significantly lower in the UCBH group relative to the CBH group [WMD = 80.57 ml, 95% CI (61.14, 99.99), *P* < 0.00001; Figure 5], with no evidence of heterogeneity (*I*^2^ = 0%, *P* = 0.89). Finally, 972 cases were included in the analysis, 476 underwent CBH and 496 underwent UCBH.

#### 3.2.3. Length of hospital stay

**FIGURE 6:**
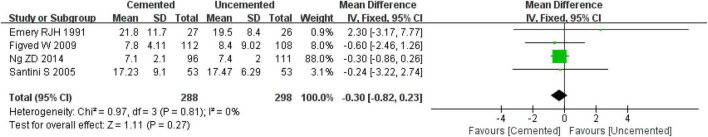
Forest plot for comparison of length of hospital stay between the cemented bipolar hemiarthroplasty (CBH) and uncemented bipolar hemiarthroplasty (UCBH) group.

A total of four studies reported the length of hospital stay, three were RCTs (28–30) and one was an observational study (38). The results comprised 586 cases, 288 underwent CBH and 298 underwent UCBH. There was no evidence of heterogeneity among the studies *(I*^2^ = 0%, *P* = 0.81). The fixed-effect model analysis showed that there was no significant difference in the length of hospital stay between two groups [WMD = −0.30 days, 95% CI (−0.82, 0.23), *P* = 0.27; Figure 6].

#### 3.2.4. Wound infections

**FIGURE 7:**
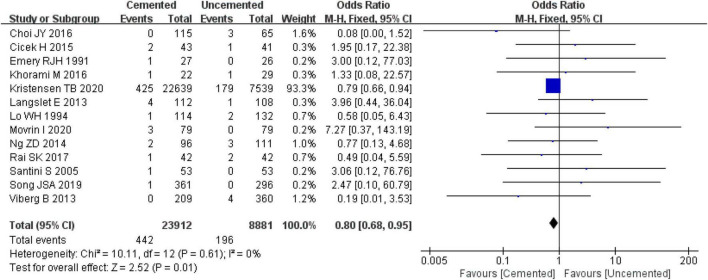
Forest plot for comparison of wound infections between cemented bipolar hemiarthroplasty (CBH) group and uncemented bipolar hemiarthroplasty (UCBH) group.

Thirteen studies reported wound infections, four were RTCs (25, 26, 29, 30) and nine were observational studies (31–39). A total of 32,793 cases were included in the analysis, 23,912 underwent CBH and 8,881 underwent UCBH. There was no evidence of heterogeneity among the studies (*I*^2^ = 0%, *P* = 0.61). Results of the fixed-effect model analysis showed that the incidence of wound infections in the CBH group was significantly lower relative to the UCBH group [OR = 0.80, 95% CI (0.68, 0.95), *P* = 0.01; Figure 7].

#### 3.2.5. Residual pain

**FIGURE 8:**
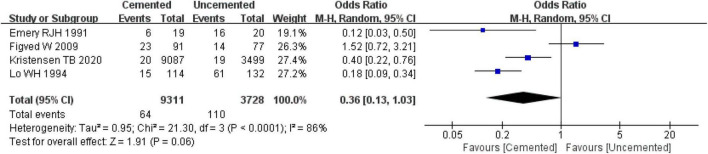
Forest plot for comparison of residual pain at last follow-up between cemented bipolar hemiarthroplasty (CBH) group and uncemented bipolar hemiarthroplasty (UCBH) group.

Three studies reported residual pain, one was RCT (28, 30) and two were observational studies (31, 39). In total of 12,589 cases were included in the analysis, 9,311 underwent CBH and 3,728 underwent UCBH. The pooled effect size analysis of the three studies revealed significantly high heterogeneity (*I*^2^ = 86%, *P* = 0.11). Next, the studies were analyzed with the random-effect model which found no significant difference between CBH group and UCBH group [OR = 0.36, 95% CI (0.13, 1.03), *P* = 0.06; Figure 8].

#### 3.2.6. Revisions

**FIGURE 9:**
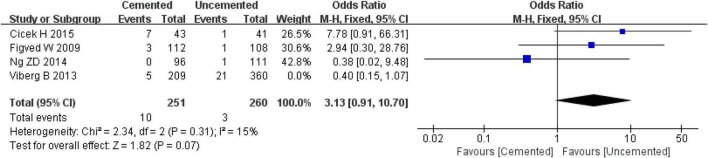
Forest plot for comparison of revision rate between cemented bipolar hemiarthroplasty (CBH) group and uncemented bipolar hemiarthroplasty (UCBH) group.

Four studies reported revisions, one was RCT (28) and three were observational studies (36–38). Pooled effect size of the four studies showed that there was high heterogeneity (*I*^2^ = 62%, *P* = 0.05). Further sensitivity analysis showed that the high heterogeneity was from the study by Viberg B et al. (38). When the study by Viberg B et al. (38) was excluded, the fixed-effect model analysis revealed no significant difference in revision rates between two groups [OR = 3.13, 95% CI (0.91, 10.70), *P* = 0.07; Figure 9], and there was no significant heterogeneity (*I*^2^ = 15%, *P* = 0.31). Finally, 511 cases were included in the analysis, 251 underwent CBH and 260 underwent UCBH.

#### 3.2.7. Re-operations

**FIGURE 10:**
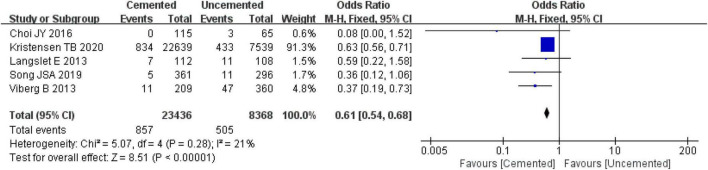
Forest plot for comparison of re-operation rates between cemented bipolar hemiarthroplasty (CBH) group and uncemented bipolar hemiarthroplasty (UCBH) group.

Five studies reported re-operations, one was RCT (26) and four were observational studies (31, 32, 34, 39). A total of 31,804 cases were included in the analysis, 23,436 underwent CBH and 8,368 underwent UCBH. There was no significant heterogeneity among the studies (*I*^2^ = 21%, *P* = 0.28). The fixed-effect model analysis showed that the re-operation rates was significant lower in the CBH group than in the UCBH group [OR = 0.61, 95% CI (0.54, 0.68), *P* < 0.00001; Figure 10].

#### 3.2.8. Complications related to prosthesis

##### 3.2.8.1. Prosthesis dislocation

**FIGURE 11:**
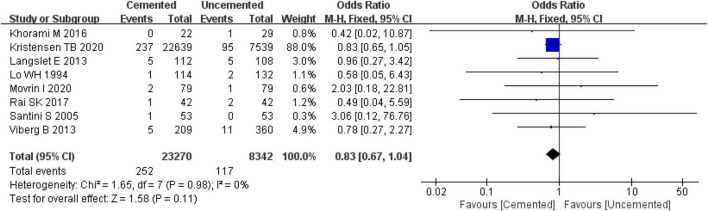
Forest plot for comparison of prosthesis dislocations between cemented bipolar hemiarthroplasty (CBH) group and uncemented bipolar hemiarthroplasty (UCBH) group.

Eight studies reported prosthesis dislocation, three were RCTs (25, 26, 29) and five were observational studies (31, 33, 35, 38, 39). A total of 31,612 cases were included in the analysis, 23,270 underwent CBH and 8,342 underwent UCBH. There was no evidence of heterogeneity among the studies (*I*^2^ = 0%, *P* = 0.98). The fixed-effect model analysis showed that there was no significant difference in the incidence of prosthesis dislocation between the two groups [OR = 0.83, 95% CI (0.67, 1.04), *P* = 0.11; Figure 11].

##### 3.2.8.2. Intra-operative fractures

**FIGURE 12:**
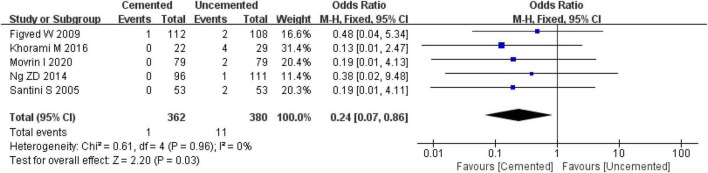
Forest plot for comparison of intra-operative fractures between cemented bipolar hemiarthroplasty (CBH) group and uncemented bipolar hemiarthroplasty (UCBH) group.

Five studies reported intra-operative fractures, three were RCTs (25, 28, 29) and two were observational studies (35, 37). A total of 742 cases were included in the analysis, 362 underwent CBH and 380 underwent UCBH. There was no evidence of heterogeneity among the studies (*I*^2^ = 0%, *P* = 0.96). Results of the fixed-effect model analysis showed that the incidence of intra-operative fractures in the CBH group was significantly lower compared with the UCBH group [OR = 0.24, 95% CI (0.07, 0.86), *P* = 0.03; Figure 12].

##### 3.2.8.3. Periprosthetic fractures

**FIGURE 13:**
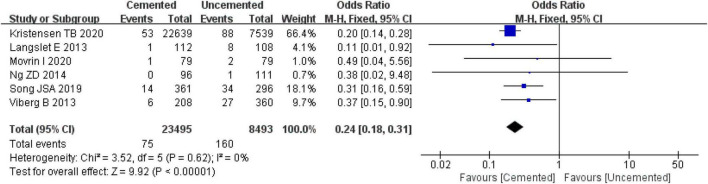
Forest plot for comparison of periprosthetic fractures between cemented bipolar hemiarthroplasty (CBH) group and uncemented bipolar hemiarthroplasty (UCBH) group.

Six studies reported periprosthetic fractures, two were RCTs (25, 26) and four were observational studies (31, 32, 37, 38). A total of 31,611 cases were included in the analysis, 23,496 underwent CBH and 8,439 UCBH. There was no evidence of heterogeneity among the studies (*I*^2^ = 0%, *P* = 0.62). Results of the fixed-effect model analysis showed that the incidence of periprosthetic fractures in the CBH group was significantly lower compared with the UCBH group [OR = 0.24, 95% CI (0.18, 0.31), *P* < 0.00001; Figure 13].

##### 3.2.8.4. Aseptic loosening of prosthesis

**FIGURE 14:**
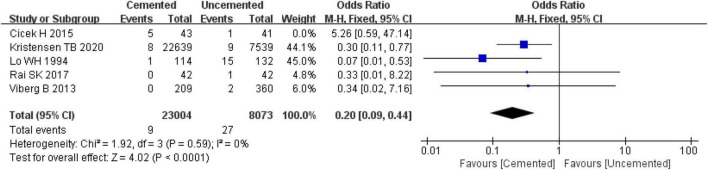
Forest plot for comparison of aseptic loosening of prosthesis between cemented bipolar hemiarthroplasty (CBH) group and uncemented bipolar hemiarthroplasty (UCBH) group.

Five observational studies reported aseptic loosening of prosthesis (31, 33, 36, 38, 39), and there was high heterogeneity among the five studies as determined by the pooled effect size (*I*^2^ = 53%, *P* = 0.08). Further sensitivity analysis revealed that the high heterogeneity was caused by the study by Cicek H et al. (36). When the study by Cicek H et al. (36) was excluded, the remaining articles were analyzed with the fixed-effect model which showed that the incidence of aseptic loosening of prosthesis was significantly lower in the CBH group compared with the UCBH group [OR = 0.20, 95% CI (0.09, 0.44), *P* < 0.00001; Figure 14], and there was no evidence of heterogeneity (*I*^2^ = 0%, *P* = 0.59). Finally, 31,077 cases included in this analysis, 23,004 underwent CBH and 8,073 underwent UCBH.

##### 3.2.8.5. Subsidence of prosthesis (>5 mm)

**FIGURE 15:**
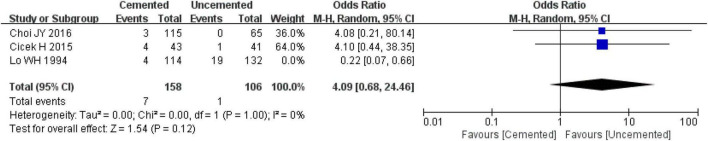
Forest plot for comparison of subsidence of prosthesis (>5 mm) between cemented bipolar hemiarthroplasty (CBH) group and uncemented bipolar hemiarthroplasty (UCBH) group.

Three observational studies reported the subsidence of prosthesis (> 5 mm) (34, 36, 39), the pooled effect size of the three studies revealed significantly high heterogeneity (*I*^2^ = 73%, *P* = 0.08), and a further sensitivity analysis showed that the heterogeneity originated from the study by Lo WH et al. (39). After excluding the study by Lo WH et al. (39), the remaining studies were analyzed with the fixed-effect model which revealed no significant difference in the incidence of subsidence of prosthesis (> 5 mm) between the two groups [OR = 4.09, 95% CI (0.68, 24.46), *P* = 0.12; Figure 15], and there was no significant heterogeneity (*I*^2^ = 0%, *P* = 1.00). Finally, 264 cases were included in the analysis, 158 underwent CBH and 106 underwent UCBH.

##### 3.2.8.6. Acetabulum erosion

**FIGURE 16:**
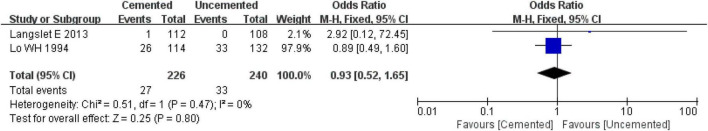
Forest plot for comparison of acetabulum erosions between cemented bipolar hemiarthroplasty (CBH) group and uncemented bipolar hemiarthroplasty (UCBH) group.

Two studies reported acetabulum erosion, one study was RCT (26) and another was an observational study (39). A total of 466 cases were included in the analysis, 226 underwent CBH and 240 underwent UCBH. There was no evidence of heterogeneity among the studies (*I*^2^ = 0%, *P* = 0.47). The fixed-effect model analysis showed there was no significant difference in the incidence of acetabulum erosion between the two groups [OR = 0.93, 95% CI (0.52, 1.65), *P* = 0.80; Figure 16].

##### 3.2.8.7. Heterotopic ossification

**FIGURE 17:**
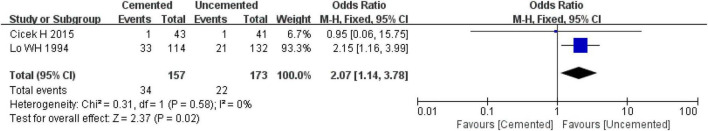
Forest plot for comparison of heterotopic ossifications between cemented bipolar hemiarthroplasty (CBH) group and uncemented bipolar hemiarthroplasty (UCBH) group.

Two observational studies reported heterotopic ossification (36, 39). The studies comprised 330 cases, 157 underwent CBH and 172 underwent UCBH. There was no evidence of heterogeneity among the studies (*I*^2^ = 0%, *P* = 0.58). The fixed-effect model for analysis showed that the incidence of heterotopic ossification in the CBH group was higher compared with the UCBH group [OR = 2.07, CI (1.14, 3.78), *P* = 0.02; Figure 17].

#### 3.2.9. General complications

**FIGURE 18:**
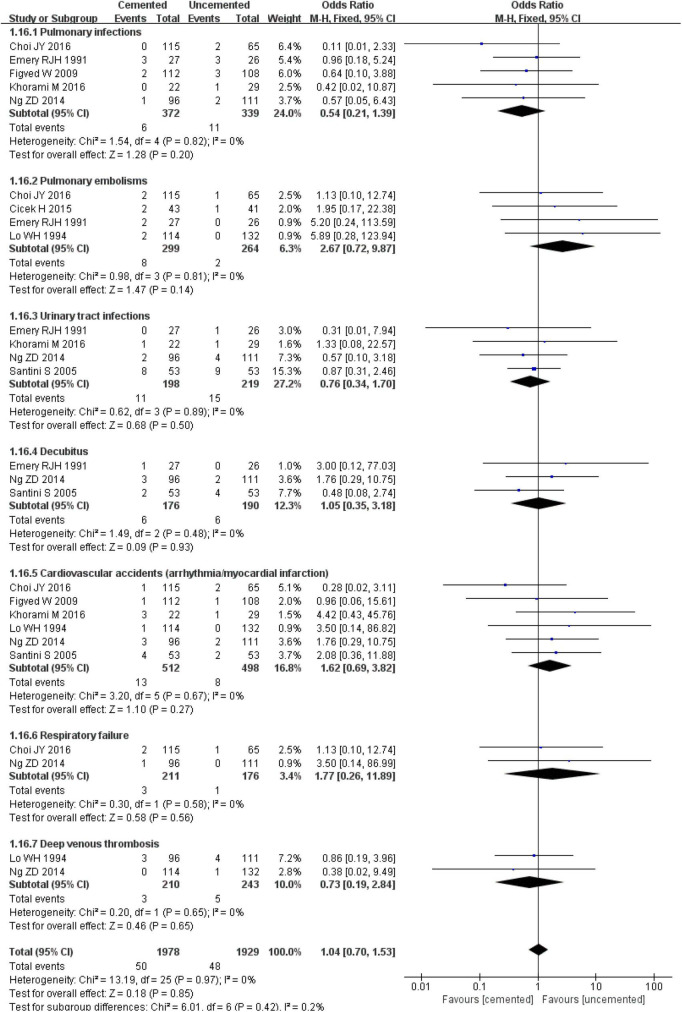
Forest plot for comparison of general complications between cemented bipolar hemiarthroplasty (CBH) group and uncemented bipolar hemiarthroplasty (UCBH) group.

The pooled effect analysis showed no evidence of heterogeneity (*I*^2^ = 0%, *P* = 0.97) among the studies in terms of pulmonary infections, pulmonary embolisms, urinary tract infections, cardiovascular accidents (arrhythmia/myocardial infarction), respiratory failure, and deep venous thromboses. In addition, there was no significant difference in the incidence of pulmonary infections [OR = 0.54, CI (0.21, 1.39), *P* = 0.20; *I*^2^ = 0%, *P* = 0.82], pulmonary embolisms [OR = 2.67, CI (0.72, 9.87), *P* = 0.14; *I*^2^ = 0%, *P* = 0.81], urinary tract infections [OR = 0.76, CI (0.34, 1.70), *P* = 0.50; *I*^2^ = 0%, *P* = 0.89], decubitus [OR = 1.05, CI (0.35, 3.18), *P* = 0.93; *I*^2^ = 0%, *P* = 0.48], cardiovascular accidents (arrhythmia/myocardial infarction) [OR = 1.62, CI (0.69, 3.82), *P* = 0.27; *I*^2^ = 0%, *P* = 0.67], respiratory failure [OR = 1.77, CI (0.26, 11.89), *P* = 0.56; *I*^2^ = 0%, *P* = 0.58], and deep venous thromboses [OR = 0.73, CI (0.19, 2.84), *P* = 0.65; *I*^2^ = 0%, *P* = 0.65] between the two groups. The incidence of general complications was not significantly different between the two groups [OR = 1.04, CI (0.70, 1.53), *P* = 0.84; *I*^2^ = 0%, *P* = 0.97; Figure 18].

#### 3.2.10. Mortality

**FIGURE 19:**
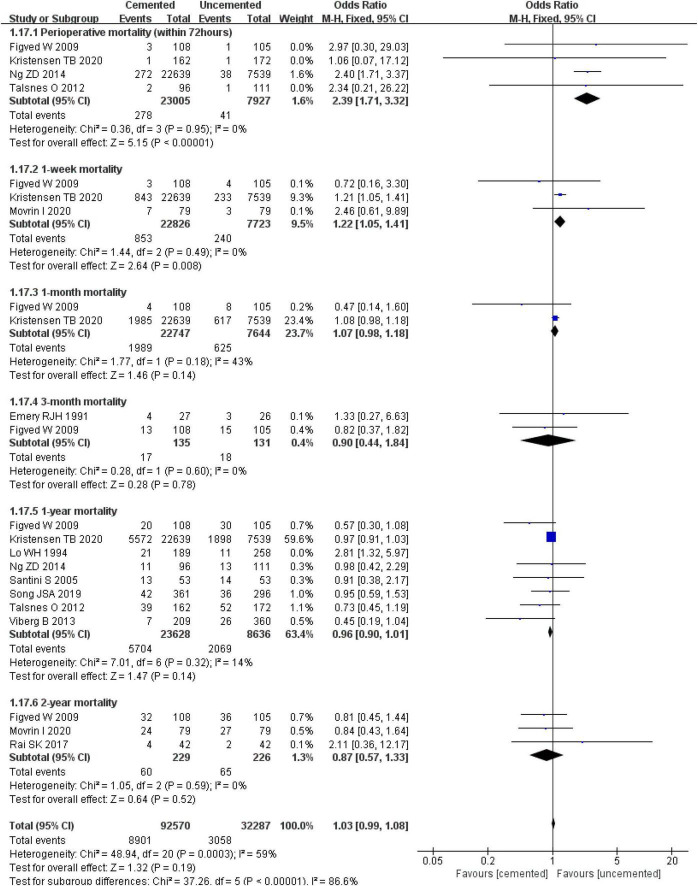
Forest plot for comparison of mortality between cemented bipolar hemiarthroplasty (CBH) group and uncemented bipolar hemiarthroplasty (UCBH) group.

The pooled effect analysis showed that perioperative mortality (within 72 h) [OR = 2.39, CI (1.71, 3.32), *P* < 0.00001; *I*^2^ = 0%, *P* = 0.95] and 1-week mortality postoperatively [OR = 1.22, CI (1.05, 1.41), *P* = 0.008; *I*^2^ = 0%, *P* = 0.49] in the CBH group were significantly lower compared with those in the UCBH group. There were no significant differences in 1-month mortality post-operatively [OR = 1.07, CI (0.98, 1.18), *P* = 0.14; *I*^2^ = 43%, *P* = 0.18], 3-month mortality post-operatively [OR = 0.90, CI (0.44, 1.84), *P* = 0.78; *I*^2^ = 0%, *P* = 0.60], 1-year mortality post-operatively [OR = 0.96, CI (0.90, 1.01), *P* = 0.14; *I*^2^ = 14%, *P* = 0.32], and 2-year mortality post-operatively [OR = 0.87, CI (0.57, 1.33), *P* = 0.52; *I*^2^ = 0%, *P* = 0.59] between the two groups (Figure 19).

### 3.3. Heterogeneity and sensitivity analysis

The results of heterogeneity and sensitivity analyses demonstrated high heterogeneity in the indicators such as intra-operative blood loss, residual pain, revision, aseptic loosening of prosthesis, subsidence of prosthesis (> 5 mm), and 1-year mortality. Further sensitivity analysis showed that there were no significant changes in the results, indicating that the heterogeneity had little influence on results and the findings were relatively reliable.

### 3.4. Publication bias

**FIGURE 20:**
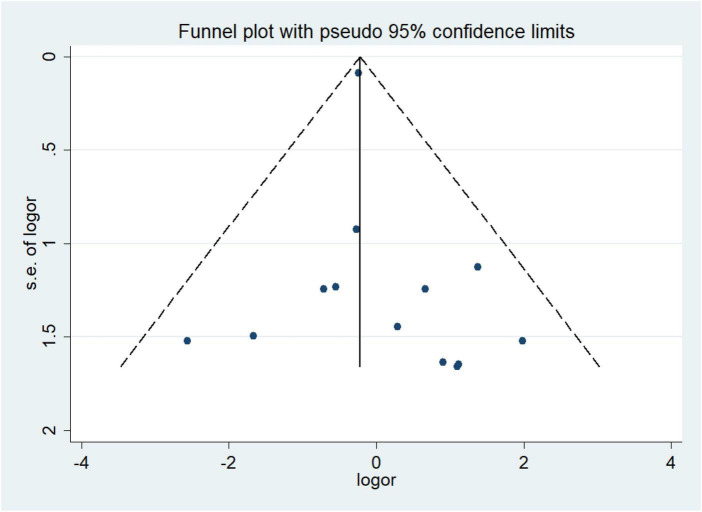
Funnel plots for publication bias assessment in wound infections.

**FIGURE 21:**
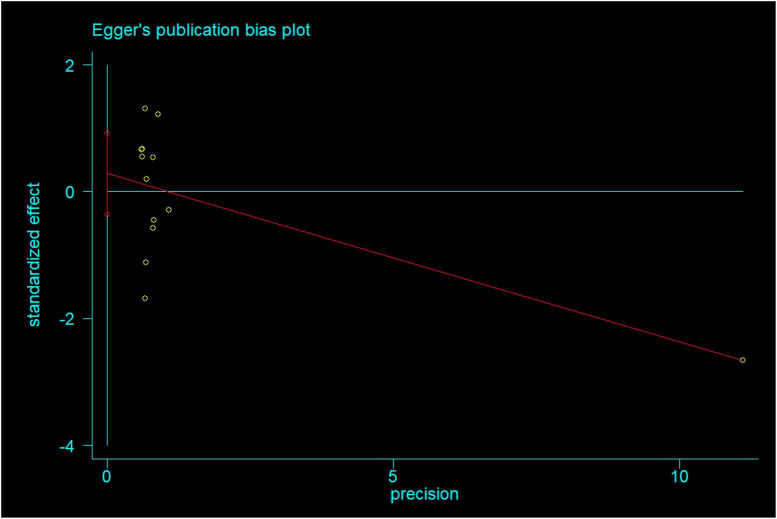
Egger’s publication bias plot in wound infections.

We constructed the funnel plot and Egger’s test for wound infections to explore the level of publication bias. Analysis of the funnel plot showed that the scatter plot was symmetrical indicating that the level of publication bias was low (Figure 20). In addition, the Egger’s test revealed that there was no evidence of publication bias in wound infections data among studies (*P* = 0.351) (Figure 21).

## 4. Discussion

Due to increased life expectancy and the resultant large elderly population, the incidence of femoral neck fractures (FNFs) is on the rise (1) as elderly patients are prone to FNFs after falling—such fractures heal slowly due to unique anatomy (4). With the continuous improvement of surgical technology, hemiarthroplasty has been successfully used to treat elderly patients ailing from FNFs. This effectively reduced the incidence of complications -such as urinary tract infection, decubitus, deep venous thrombosis, and hypostatic pneumonia—caused by long-term bed rests following traditional treatments (3). A bipolar head has double-action features—the joint activity is concurrently operated by both internal and external joints—which not only reduced the wear of the acetabulum but also greatly extended the service life of the prosthesis (6, 7). A meta-analysis showed the rate of acetabular erosion in bipolar hemiarthroplasty was significantly lower than that in unipolar hemiarthroplasty (1.2 and 5.5%, respectively), yet the other compared outcomes showed no significant difference between two groups (8). Bipolar hemiarthroplasty remains the main current surgical method for the treatment of FNFs in the elderly. Furthermore, the use of cemented or non-cemented prostheses in treating FNFs in elderly patients remains debatable. Most meta-analyses that compared cemented and uncemented hemiarthroplasty in treating FNFs included both bipolar and unipolar head prostheses. Therefore, our meta-analysis compared the effectiveness and safety between cemented and uncemented hemiarthroplasty for the treatment of FNFs in elderly patients only for bipolar head prostheses. Elmenshawy AF et al. (10) compared cemented bipolar hemiarthroplasty (CBH) and uncemented bipolar hemiarthroplasty (UCBH) for FNFs treatment in the elderly, but the included literature which were published before 2014. Another meta-analysis compared CBH and UCBH was for the treatment of unstable intertrochanteric fractures (9).

We found that CBH associated with longer operation time, considering the reasons was that the cemented hemiarthroplasty need to prepare and place the bone cement, as well as waiting for the cement to set. In theory, the prolonged operation time may result in increasing intra-operative blood loss during operation, this hypothesis was consistent with our result that CBH associated with more intra-operative blood loss in comparison to UCBH. The other reason was that this higher intra-operative bleeding may in CBH also result from repeated reaming during operation that results in increasing intramedullary hemorrhage. However, intra-operative blood loss from cemented and uncemented hemiarthroplasty did not significantly differ in a previous meta-analysis as although cemented hemiarthroplasty had longer operation durations, the bone cement immediately closed the medullary cavity, thus reducing intra-operative blood loss (40). Kong XG (41) found that postoperative hospital stays were shorter for patients who underwent cemented hemiarthroplasty than uncemented hemiarthroplasty. He hypothesized that a cemented prosthesis achieves good stability in the early postoperative period, since bone cement fixation filled the gap between the trabecular bone and prosthesis with bone cement, creating a micro internal locking fixation at the interface of bone-bone cement-prosthesis after bone cement setting. Early stabilization of cemented prostheses promoted early out-of-bed activities and fast recoveries, which ultimately shortened postoperative hospital stays. Uncemented prostheses mainly depend on the growth of the bone tissue into the prosthesis to form a close biological fixation between bone and prosthesis surfaces, however, this takes long durations and has poor early stability (41). Since elderly patients often have a degree of osteoporosis, those with uncemented hemiarthroplasty should postpone the full weight-bearing time to avoid sinking the biological femoral stem. Our meta-analysis showed there was no significant difference in postoperative hospital stays between patients who had underwent CBH and UCBH procedures.

Surgical wound infection is a tabooed complication for surgeons, and increased operation time theoretically increase the risk of wound infection. There was a lower association with the incidence of wound infections for CBH than UCBH as per our meta-analysis which had a pooled effect size of 13 studies and no significant publication bias (Egger’s test, *P* = 0.351). Wound infection included superficial and deep infection. Four studies reported deep wound infections (25, 26, 29, 39), two reported superficial wound infections (26, 39), and the other nine reported unspecified wound infections (30–38). Therefore, these were all summarized as wound infections in the meta-analysis. Deep wound infection following hip arthroplasty was a disastrous problem, especially for highly susceptible elderly patients. When we only included deep wound infections in our meta-analysis, there was no significant difference between CBH and UCBH corroborating the findings of Wu XJ et al. (40). Likewise, there was no significant difference in superficial wound infections between CBH and UCBH (40). The meta-analysis of Sebastian S et al. (42) showed that antibiotic (gentamicin)-containing bone cement effectively reduced the incidence of prosthetic joint infections following hip arthroplasty. However, in our meta-analysis, the included studies did not indicate whether the bone cement contained antibiotics. Wound infection after a hip arthroplasty was not only related to the operation time and the use of bone cement supplemented with antibiotics but also risk factors such as prolonged wound drainage, glucocorticoids, poor glycemic control, urinary/respiratory infections, chronic liver disease, and alcohol consumption (43). Clinically, the plan of hip replacement should include relevant prevention and control measures—detailed preoperative physical examinations to exclude possible hidden infectious lesions, strict intra-operative aseptic operation, and standardized postoperative nursing—to not only reduce incidences of postoperative wound infection but also improve the treatment effect.

Bone cement implantation syndrome (BCIS) is a set of clinical symptoms caused by bone cement implantation that include hypoxia, sudden drop of arterial pressure, pulmonary hypertension, arrhythmias, loss of consciousness, and cardiac arrest (16). The pathophysiology of BCIS is not clear, but bone cement toxicity, pulmonary embolisms, and lipid mediators are theorized to constitute its etiology. Moreover, physiological reactions and original pathological changes in patients are involved in its pathogenesis (16). BCIS is a potentially fatal complication of cemented hemiarthroplasty and confers a 16-fold increased chance of death within the 30-day postoperative period (16). Analysis of 25,000 hemiarthroplasties from the Australian registry (44) found that the early postoperative mortality of patients was significantly higher for those who had underwent cemented than uncemented hemiarthroplasties, and the difference decreased with time. And they also found that patients older than 80 years of age had an increased risk of death after undergoing a cemented hemiarthroplasty. An analysis of 11,210, 19,669, and 25,174 hemiarthroplasties from the Norwegian (45), UK (46), and Finland registries (47) respectively found a higher first-day post-operation mortality for patients who had underwent cemented than uncemented hemiarthroplasties. Thus, for elderly patients, the use of the traditional bone cement hemiarthroplasty increases early postoperative mortality. In our meta-analysis, the perioperative mortality (within 72 h after operation) and 30-day postoperative mortality were significantly higher for CBH than UCBH. However, there were no significant differences in postoperative mortality between the two groups at 1 month, 3 months, 1 year, and 2 years. Wu XJ et al. (40) showed an association with higher mortality at 1 day, 1 week, and 1 month after operation for cemented than uncemented hemiarthroplasty. Langslet E et al. (26) found no significant difference in mortality at 5 years between the cemented and uncemented groups, but more high-quality studies are needed to corroborate this finding. Relatedly, Olsen F et al. (16) reported high American Society of Anesthesiologists (ASA) grade, chronic obstructive pulmonary disease, and medication with diuretics and warfarin as the independent preoperative risk factors for the development of BCIS and severe BCIS was associated with high early and late mortalities. Therefore, we should think about how to reduce the occurrence of BCIS in clinical work so as to reduce the mortality of patients. The application of bone cement is only just one of many factors that predispose the patient to postoperative mortalities. Most patients with FNFs are at an advanced age and in a generally poor condition often ailing from cardiovascular and cerebrovascular diseases, hypertension, diabetes, and lung diseases and low physical reserves, those are risk factors for higher mortality, demonstrating the importance of perioperative management of patients (48).

Cardiovascular and cerebrovascular accident complications and pulmonary embolisms were the main concerns for patients undergoing cemented hemiarthroplasty. Insertion of the prosthesis stem into the medullary cavity using the cemented technique, raised the intramedullary pressure, which accentuated fat embolization (49). Fat embolization caused hemodynamic imbalance and embolism of important organs, resulting in cerebral and myocardial infarctions, pulmonary embolisms, acute heart and respiratory failure, shock, and even sudden death both during operation and postoperatively (50). Mori K et al. (51) reported the sudden death of a patient due to severe fat embolization in the lung 4 h after the operation. Movrin I (25) showed that patients undergoing cemented than uncemented hemiarthroplasty were more likely to have a sudden drop in systolic blood pressure (≥ 30 mmHg) and in intraoperative SaO2 during the prosthesis stem insertion (18.9 vs. 5.1%, *P* = 0.007; 10.1 vs. 0.0%, *P* = 0.009). Relatedly, on study reported a patient experienced severe hypotension during the cementing procedure and died within 24 h of myocardial infarction (28). Therefore, some scholars believe that the elderly patients with significant cardiovascular risk factors should be given priority to the use of cementless bipolar hemiarthroplasty (52). A previous meta-analysis showed that the cemented hemiarthroplasty did not increase the risk of mortality, cardiovascular and cerebrovascular complications while achieving good postoperative results (53). Our meta-analysis obtained similar results that there were no significant differences in the incidence of pulmonary embolisms, cardiovascular accidents (arrhythmia/myocardial infarction), and respiratory failure between CBH and UCBH. In summary, there were several points that deserved mention when using bone cement, attention should be paid to the maintenance of the patient’s blood volume at a normal level during anesthesia; stable blood pressure before using bone cement, and heightened monitoring during injection; before reaming or use of bone cement, flushing of the intramedullary contents with a pulsing squirt gun and ensuring hemostasis *via* gauze compression to reduce the pressure in the medullary cavity; the appropriate speed of bone cement injection. In addition, our meta-analysis showed there were no significant differences between CBH and UCBH in incidences of other general complications such as pulmonary infections, urinary tract infections, deep venous thromboses, and decubitus. Both CBH and UCBH promoted get-out-of-bed activities early and reduced the incidence of bed-related complications.

The revision rates of prostheses is the most convincing current index for evaluation of the failure of hip arthroplasty. Deep infection, loosening of prosthesis, and femoral fracture lead to the failure of hip arthroplasty that in turn leads to revision surgery. Analysis of 11,116 patient records from the Norwegian Hip Fracture Registry found higher revision rates in the ucemented group than in the cemented group (54). Our meta-analysis found no significant difference in revision rates between CBH and UCBH. However, CBH was associated with lower incidences of intra-operative fractures, periprosthetic fractures after an operation, aseptic loosening of the prosthesis, and deep infections. Re-operation included revision that were mainly for periprosthetic fracture, aseptic loosening of the prosthesis, deep infection, dislocation of prosthesis, and wear of acetabular. However, re-operation also included the reduction of a dislocated prosthesis, debridement of superficial wound infection, and drainage of hematoma. Our meta-analysis found an association of lower re-operation rates for CBH than UCBH, corroborating the findings of Wu XJ et al. (40) and Gjertsen JE et al. (54). In our research, this lower re-operation rates may relate to lower incidences of wound infections. The stability of uncemented prosthesis depends on the contact scope and close degree between prosthesis and medullary cavity. Otherwise, resulting in either loosening or subsidence of the prosthesis results in the near future or in the long term. Elderly patients often ail from severe osteoporosis, and to stabilize the prosthesis, the surgeons constantly knock the prosthesis stem to tamp the prosthesis. This may lead to fracturing of the upper femur intra-operatively. Although the initial stability of uncemented prosthesis depends on well-matched bone tissue and prosthesis, it is difficult for elderly patients with severe osteoporosis to achieve this goal. This leads to increased chances of a loose prosthesis that poorly disperses the surrounding stress leading to periprosthetic fracture during weight-bearing walking (41). Therefore, accurate preoperative template measurement, careful surgical steps without the usage of major force, careful assessment of bone quality, and selection of appropriate prosthesis are extremely important for the treatment of FNFs in the elderly using hemiarthroplasty. Our meta-analysis showed no significant differences between CBH and UCBH in other prosthesis-related complications including dislocation of prosthesis, subsidence of prosthesis (> 5 mm), and acetabulum erosion. Kizkapan TB et al. (55) showed that bone cement usage does not affect the risk of dislocation after bipolar hemiarthroplasty, which was mainly related to the surgical approach and pelvic morphologic features. Age, placement position of the prosthesis, neurological disorders, abductor muscle weakness, hip joint deformities, the diameter of the artificial femoral head, and inappropriate posture are the other risk factors for the dislocation of prostheses (41, 55). Acetabular wear is a problem for hemiarthroplasty and thus total hip arthroplasty is the first option for patients younger than 70 years of age, although there is no clear age limit for use of either hemiarthroplasty or total hip arthroplasty. Moreover, it also needs to be comprehensively considered in combination with the patient’s physical condition and daily activity. Thigh pain often occurs early on after an uncemented hip arthroplasty (56), which may be related to the fretting of prosthesis in the early stage and the proportion of the prosthesis in the medullary cavity after implantation (57). Conversely, cemented fixation is associated with a low incidence of thigh pain due to the immediate stability of the prosthesis in the early stage. Our meta-analysis showed no significant difference between CBH and UCBH in the incidence of residual pain. This was because the assessment of thigh pain in the included studies happened at the last follow-up—by this time, the bone tissue had grown well into the uncemented prosthesis and achieved ultimate stability. This was corroborated by Campbell et al. (57) who showed that thigh pain was relieved with time when the uncemented prosthesis achieved a further fit with the medullary cavity. This indicated a reduced incidence of thigh pain when the uncemented prosthesis matched the medullary cavity well. Therefore, the selection of a fitting prosthesis greatly influences the prevention of thigh pain. Currently, a hydroxyapatite-coated femoral prosthesis has characteristics that better promote bone induction and bone ingrowth (58). Some studies (57, 59) showed that the interface between hydroxyapatite and the bone was combined firmly 3 weeks after hip arthroplasty, resulting in a sufficient and uniform fixation of the prosthesis in the medullary cavity. Heterotopic ossification is a common complication of hip arthroplasty. Although most of the patients have no obvious clinical symptoms, severe heterotopic ossification causes postoperative pain and decreased joint mobility (60). The pathogenesis of heterotopic ossification is unclear: injuries to the muscles, nerves, and other soft tissues during operation possibly transform fibroblasts to osteoblasts, resulting in heterotopic ossification (61). Our meta-analysis showed an association with a lower incidence of heterotopic ossification for UCBH than CBH, possibly because both the heat released by the polymerization of the bone cement and its cytotoxicity may stimulate the formation of heterotrophic ossification in the surrounding tissues (40). Non-steroid anti-inflammatory drugs can prevent the formation of heterotopic ossification (62). Therefore, the incidence of heterotopic ossification can be reduced by light and soft manipulations during operation to avoid excessive soft tissue damage, thorough flushing of the wound surface before closing the incision, and the preventive use of non-steroidal anti-inflammatory drugs after the operation.

## 5. Limitation

Our study had some limitations. First, there only six RCTs were included, and the rest were observational studies, which creating an inevitable bias. Second, it was difficult to strictly control for the blinding to the limitations of surgery. Therefore, better research plans, larger sample sizes, more multi-center RCTs, and longer follow-up durations are recommended for future meta-analyses. Third, all the included studies were published in English, resulting in possible language biases. Fourth, the size of the medullary cavity, the thickness of the bone cortex, and the severity of osteoporosis are important factors that affect the selection of prostheses for the treatment of FNFs in elderly patients. However, our meta-analysis did not analyze these factors, but only compared outcomes between CBH and UCBH. Finally, the general status, prosthesis type and surgical approach used on the patients differed, and coalesced results should thus be treated with caution.

## 6. Conclusion

In conclusion, patients with FNFs who were older than 60 years of age and underwent CBH were associated with longer operation time, more intra-operative blood loss, higher incidence of heterotrophic ossification and higher mortality within 72 h and 1-week after operation, but had lower incidences of periprosthetic fractures, aseptic loosening of prostheses, intra-operative fractures, postoperative wound infections, and re-operations than those who underwent UCBH. Conversely, the two groups did not significantly differ in terms of length of hospital stay, residual pain, dislocation of prosthesis, subsidence of prosthesis (> 5 mm), acetabulum erosion, revision rates, incidences of pulmonary infections, pulmonary embolisms, urinary tract infections, deep venous thromboses, decubitus, cardiovascular accidents (arrhythmia/myocardial infarction), respiratory failure, mortality at 1 month, 3 months, 1 year, and 2 years postoperatively. However, due to the limited numbers and types of included studies, the above conclusions need to be corroborated by numerous RCTs with large sample sizes and long-term follow-ups.

## Data availability statement

The original contributions presented in this study are included in the article/Supplementary material, further inquiries can be directed to the corresponding authors.

## Author contributions

MF and JW designed the project and prepared the manuscript. JS, MF, ZR, and YL were involved in data collection and data analysis. MF and WJ edited the manuscript. All authors read and approved the final manuscript.
