# Supplementary material for: A systematic review and meta-analysis of cemented and uncemented bipolar hemiarthroplasty for the treatment of femoral neck fractures in elderly patients over 60 years old
Source: Front Med (Lausanne). 2023 Feb 2;10:1085485. doi: 10.3389/fmed.2023.1085485 (PMC9932906; doi:10.3389/fmed.2023.1085485)
Supplement: Supplementary file 1 [file Table_1.DOCX]

**Supplemental table 1. Search strategy for identification of studies to be included in the review**

| **Search strategy**  #1 ("Hemiarthroplasty"[Mesh] OR "Hemiarthroplasties"[Title/Abstract] OR "Hemi-Arthroplasty"[Title/Abstract] OR "Hemi Arthroplasty"[Title/Abstract] OR "Hemi-Arthroplasties"[Title/Abstract] OR "hemiprosthesis"[Title/Abstract] OR "hemiprosthesis"[Title/Abstract] OR "artifcial femoral head replacement"[Title/Abstract] OR "artifcial femoral head arthroplasty" OR "Arthroplasty" OR "Replacement")  #2 ("Bipolar"[Title/Abstract])  #3 ("Bone cements"[Mesh] OR "Cement"[Title/Abstract] OR "Cemented"[Title/Abstract])  #4 ("Uncement"[Title/Abstract] OR "Uncemented"[Title/Abstract] OR "Cementles"[Title/Abstract] OR "Without bone cement"[Title/Abstract] OR "Non-cemented"[Title/Abstract])  #5 (#1 AND #2 AND #3 AND #4) |
| --- |

1)249 articles were identified: PubMed- 65, Cochrane- 11, Embase- 50, Web of science- 123

2)157 relevant citations screened by title and abstract

3)51 articles assessed for eligibility

4)14 articles were included for meta-analysis after removing 37 articles with reasons by reading full-text.

5)the other 1 article was included after assessing the reference lists of relevant reviews
