# Supplementary material for: A systematic review and meta-analysis of cemented and uncemented bipolar hemiarthroplasty for the treatment of femoral neck fractures in elderly patients over 60 years old
Source: Front Med (Lausanne). 2023 Feb 2;10:1085485. doi: 10.3389/fmed.2023.1085485 (PMC9932906; doi:10.3389/fmed.2023.1085485)
Supplement: Supplementary file 2 [file Table_2.pdf]

Supplementary table 2. Author's judgements about each risk of bias for each included RCTs based on Cochrane risk of bias assessment items

Included RCTs	Random sequence generation	Allocation concealment	Blinding of participants and personnel	Blinding of outcome assessment	Incomplete outcome data	Selective reporting	Other bias	Quality grade
Movrin I et al. (2020)	+	+	?	?	+	+	+	B
Langslet E et al. (2012)	+	+	+	+	+	+	+	A
Talsnes O et al. (2012)	+	+	+	+	+	+	?	B
Figved W et al. (2009)	+	+	+	+	+	+	+	A
Santini S et al. (2005)	-	-	?	?	?	+	?	C
Emery RJH et al. (1991)	+	+	?	?	+	+	+	B

"+": low risk of Bias, "-": high risk of bias; "?": unclear.

"A": high quality; "B": moderate quality; "C": low quality
