# Supplementary material for: A systematic review and meta-analysis of cemented and uncemented bipolar hemiarthroplasty for the treatment of femoral neck fractures in elderly patients over 60 years old
Source: Front Med (Lausanne). 2023 Feb 2;10:1085485. doi: 10.3389/fmed.2023.1085485 (PMC9932906; doi:10.3389/fmed.2023.1085485)
Supplement: Supplementary file 3 [file Table_3.pdf]

Supplementary table 3. Author's judgements about each risk of bias for each included observational studies based on Newcastle Ottawa scale

	Selection (4 scores)				Comparability	Result (3 scores)			Total scores	Quality grade
	A	B	C	D	(2 scores)	E	F	G	(9 scores)	
Kristensen TB et al. (2020)	1	1	1	1	1	1	1	1	8	High
Song JSA et al. (2019)	1	1	1	0	1	1	1	0	6	Moderate
Rai SK et al. (2017)	1	1	1	1	1	1	1	0	7	High
Choi JY et al. (2016)	1	1	1	1	1	1	1	1	8	High
Khorami M et al. (2016)	1	1	1	1	2	1	1	0	8	High
Cicek H et al. (2015)	1	1	1	1	1	0	1	1	7	High
Ng ZD et al. (2014)	1	1	1	1	1	0	1	1	7	High
Viberg B et al. (2013)	1	1	1	0	2	1	1	0	7	High
Lo WH et al. (1994)	0	1	1	1	1	1	1	1	7	High

Note: A, representativeness of the exposed cohort: B, selection of the non-exposed cohort; C, ascertainment of exposure; D, demonstration that outcome of interest was not present that at the start of study; E, assessment of outcome; F, follow-up long enough for outcomes to occur; G, adequacy of follow-up of cohort
